# Global Research Landscape and Emerging Trends in the Treatment of Female Chronic Pelvic Pain: A Comprehensive Bibliometric Analysis (2005–2026)

**DOI:** 10.1155/prm/7732443

**Published:** 2026-10-18

**Authors:** Zheng Hou, Ying Yao, Haojie He, Sha Wang, Yang Chen

**Affiliations:** ^1^ Department of Obstetrics and Gynecology Peking University Third Hospital Beijing 100191 China puh3.net.cn; ^2^ National Clinical Research Center for Obstetrics and Gynecology (Peking University Third Hospital) Beijing 100191 China

**Keywords:** bibliometric analysis, chronic pelvic pain, female, research trends, treatment

## Abstract

**Purpose:**

This study aimed to conduct a comprehensive bibliometric analysis to characterize global research patterns, scientific output, and emerging trends in the treatment of female chronic pelvic pain (CPP).

**Methods:**

Publications related to female CPP treatment were retrieved from the Web of Science Core Collection (WoSCC), limited to the Science Citation Index Expanded (SCIE) and Social Sciences Citation Index (SSCI). The search covered January 2005 to June 2026 and was restricted to English‐language original articles. An expanded Boolean search strategy incorporating terms for population, condition, and intervention was applied. The retrieval was completed on June 29, 2026. Bibliometric indicators were analyzed using VOSviewer (v.1.6.20), CiteSpace (v.6.3.R1), and the R package bibliometrix (v.4.3.3). Author, institution, and country names were standardized prior to analysis.

**Results:**

A total of 14,110 articles were identified, contributed by 57,872 authors from 41,677 institutions across 123 countries, published in 1828 journals. The annual growth rate was 8.44% (complete calendar years 2005–2025). The USA led in publication volume (*n* = 2916) and total citations (TC = 91,126). Harvard University was the most productive institution (814 articles). *Fertility and Sterility* ranked first in within‐dataset h‐index (94) and TC = 34,864. Keyword co‐occurrence analysis revealed four thematic clusters encompassing clinical assessment and psychosocial dimensions, molecular pathogenesis, surgical management, and specific populations. Citation burst analysis identified the keywords with the strongest bursts in successive periods, including “peritoneal fluid” (strength = 29.14, 2005–2013), “trial” (28.26, 2010–2018), “anxiety” (22.19, 2020–2026), and “Mendelian randomization” (17.20, 2024–2026).

**Conclusion:**

Global research on female CPP treatment has demonstrated sustained growth, with recent trends suggesting increasing emphasis on psychosocial dimensions, integrated care approaches, and novel causal inference methodologies.

## 1. Introduction

Chronic pelvic pain (CPP) is a prevalent and debilitating condition defined as persistent or recurrent pain perceived to originate in the pelvis, lasting for at least 6 months [1]. CPP affects up to 26% of women worldwide and is associated with substantial impairment in physical, psychological, and social functioning [2]. The etiology of CPP is multifactorial, encompassing gynecological disorders such as endometriosis and adenomyosis, urological conditions including interstitial cystitis, gastrointestinal pathologies, musculoskeletal dysfunction, and psychological components [3]. This complexity poses significant challenges for diagnosis and management, often necessitating comprehensive and individualized therapeutic strategies [4].

Treatment approaches for female CPP span a broad spectrum. Pharmacological options include analgesics, hormonal therapies, and neuromodulatory agents, although individual response varies considerably and many patients report only partial symptom relief [5]. Surgical interventions, ranging from laparoscopic excision of endometriotic lesions to neuromodulation procedures, are typically considered when conservative management proves insufficient [6]. In recent years, nonpharmacological conservative therapies, including pelvic floor physical therapy, psychological interventions such as cognitive behavioral therapy, and mindfulness‐based approaches, have gained increasing recognition as integral components of CPP care [7]. Current clinical guidelines, including the 2024 Society of Obstetricians and Gynaecologists of Canada (SOGC) guideline [8] and the 2022 European Society of Human Reproduction and Embryology (ESHRE) guideline [9], emphasize a biopsychosocial framework and advocate for multidisciplinary management that addresses both physical and psychological dimensions of pain.

Research output on female CPP treatment has grown substantially in recent years, with an expanding literature documenting therapeutic innovations, outcome measures, and patient‐centered approaches [10, 11]. However, despite this growth, there remains a lack of comprehensive quantitative analyses that systematically map the global research landscape in this domain. Existing studies have typically focused on specific interventions, particular patient subgroups, or narrow aspects of CPP management, leaving a gap in understanding the overall structure, collaboration patterns, and evolving thematic priorities of the field [12, 13]. A recent bibliometric study examined endometriosis pain treatment using experimental models but was limited to a small corpus of 30 publications [14]. No large‐scale bibliometric analysis has comprehensively characterized the treatment‐oriented literature on female CPP across multiple dimensions.

Bibliometric analysis offers a systematic, quantitative approach to evaluating academic literature by mapping publication trends, citation networks, collaboration patterns, and emerging research hotspots [15, 16]. By applying bibliometric methods to the field of female CPP treatment, it is possible to identify key contributing countries, institutions, authors, and journals, as well as to detect thematic shifts over time through keyword co‐occurrence and citation burst analyses [17]. This study aimed to conduct a comprehensive bibliometric analysis of global publications from 2005 to 2026 to characterize the research landscape, identify major thematic clusters, and detect emerging trends in the treatment of female CPP, thereby providing an evidence‐based foundation for guiding future research priorities and clinical practice development.

## 2. Materials and Methods

### 2.1. Search Strategy and Data Collection

A comprehensive literature search was conducted using the Web of Science Core Collection (WoSCC), which provides access to high‐quality peer‐reviewed publications across multiple disciplines [17]. The search string was TS = ((Female∗ OR Woman OR Women OR Girl∗ OR Gynecolog∗) AND (“Chronic pelvic pain∗” OR “endometriosis‐associated pain” OR “Pelvic pain∗” OR “CPP” OR “Pelvic ache∗” OR “Pelvic discomfort∗” OR “Dysmenorrhea” OR “Dyspareunia” OR “Endometriosis”) AND (Treat∗ OR Therap∗ OR Manag∗ OR Intervent∗ OR Rehabilitat∗ OR Surg∗ OR Medicat∗ OR Drug∗ OR Pharmac∗ OR Physiotherap∗)). Straight double quotation marks were used consistently for all condition terms to denote exact‐phrase searching, and the asterisk denotes truncation. The search was limited to the Science Citation Index Expanded (SCIE) and the Social Sciences Citation Index (SSCI) within the WoSCC. Only English‐language publications were included; non‐English records (*n* = 686) were excluded. The publication timeframe was restricted from January 1, 2005, to June 29, 2026. Document types were limited to original research articles; reviews (*n* = 3525), editorial materials (*n* = 507), meeting abstracts (*n* = 210), letters (*n* = 43), and other nonarticle document types (*n* = 1003) were excluded. Document type filtering relied solely on the Web of Science “Document Types” field, and only records indexed as “Article” were retained; accordingly, a small number of records that are clinical guidelines or clinical practice reviews in content but are indexed as “Article” in Web of Science remained in the dataset. Literature retrieval was completed on June 29, 2026, in a single day to ensure temporal consistency and minimize discrepancies arising from database updates. After screening, a total of 14,110 articles were included in the final analysis.

Bibliographic records were exported in “Full Record and Cited References” and “Plain Text” formats. Exported data included publication and citation metrics, article titles, author affiliations, institutional affiliations, geographic distribution, keywords, and journal sources. Prior to analysis, author names, institutional affiliations, and country names were standardized to address potential inconsistencies. For example, variant institutional entries such as “University of Michigan” and “University of Michigan System” were harmonized to avoid duplication and ensure accurate ranking. Residual variants were additionally merged manually; for example, “Univ Roma La Sapienza” and “Sapienza Univ Rome” were combined into “Sapienza University of Rome,” and all tables and figures were regenerated after this harmonization.

### 2.2. Statistical Analysis

Data visualization and analyses were performed using three complementary bibliometric tools: VOSviewer (v.1.6.20), CiteSpace (v.6.3.R1), and the R package bibliometrix (v.4.3.3). VOSviewer was used to construct co‐occurrence and bibliographic coupling networks of authors, institutions, countries, journals, and keywords, enabling identification of collaboration patterns and interdisciplinary linkages [18]. CiteSpace was employed for keyword burst detection to identify emerging research trends. Time slicing was set from January 2005 to June 2026 in 1‐year intervals, with keywords designated as the node type. A minimum threshold of five nodes per time slice was applied, and network pruning was performed using the Pathfinder and Clip‐Merge algorithms [19]. The R package bibliometrix was used to map global research distribution, assess influence metrics, and identify key trends [20].

Researcher and journal impact were quantified using the h‐index [21], which balances productivity and citation impact. The g‐index [22] was additionally incorporated to assign greater weight to highly cited publications, and the m‐index [23] normalizes the h‐index by the duration of a researcher’s publishing career, serving as an indicator of sustained scholarly productivity. Journal prestige was evaluated using 2025 Journal Citation Reports (JCR) quartile rankings (Q1–Q4) and Journal Impact Factor (IF) values. All h‐index, g‐index, and m‐index values reported in this study were calculated within the retrieved dataset and therefore differ from whole‐journal or whole‐career indices reported by other platforms. Annual publication trends were fitted by linear regression using complete calendar years only (2005–2025); the partial year 2026 (January 1 to June 29) is presented for completeness but was excluded from the regression and from the annual growth rate calculation. Keyword synonyms were also standardized prior to co‐occurrence analysis to ensure analytical accuracy.

## 3. Results

### 3.1. An Overview of Publications

After systematic screening (Figure 1), this study encompassed 14,110 publications, all classified as research articles. A total of 22,720 records were initially retrieved from the WoSCC. After excluding records published before 2005 (*n* = 2636), reviews (*n* = 3525), editorial materials (*n* = 507), letters (*n* = 43), meeting abstracts (*n* = 210), non‐English publications (*n* = 686), and other document types (*n* = 1003), 14,110 articles were retained for analysis. These articles appeared in 1828 journals spanning the period from 2005 to 2026. A total of 57,872 authors affiliated with 41,677 institutions across 123 countries contributed to these publications. The mean document age was 8.23 years, with an average citation count of 24.38 per article. The average number of co‐authors per document was 6.25, and the rate of international co‐authorship was 16.68%. The annual growth rate calculated over complete calendar years (2005–2025) was 8.44%.

**FIGURE 1:**
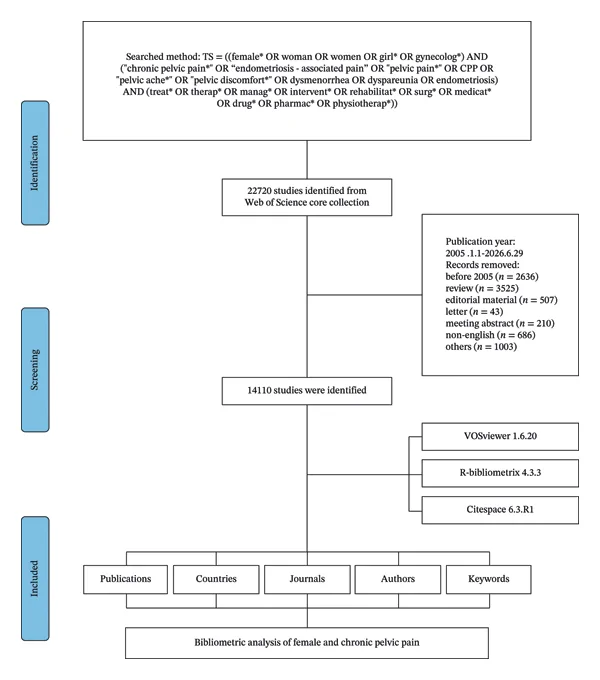
Flowchart of the literature screening process for studies on female chronic pelvic pain (CPP).

The annual publication output demonstrated a consistent upward trajectory (Figure 2). Starting with 242 articles in 2005, the field showed steady growth throughout the study period. Publication volume surpassed 600 articles annually from 2014 onward (*n* = 616) and continued to increase, reaching 692 in 2019. A notable acceleration occurred post‐2020, with annual output exceeding 900 articles (*n* = 956 in 2021). The peak was observed in 2025 with 1223 articles. The year 2026 recorded 672 articles through June 29, representing an incomplete year. Linear regression restricted to complete calendar years (2005–2025) revealed a strong positive trend, and the partial year 2026 was excluded from this analysis. The mean citation rate per article per year remained relatively stable, ranging from 2.03 to 3.18 across full publication years, indicating sustained scholarly impact.

**FIGURE 2:**
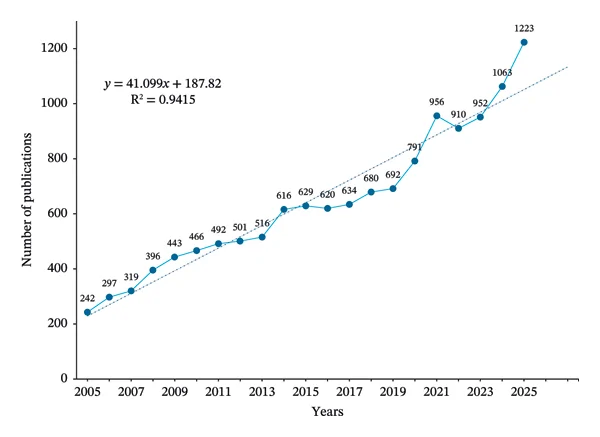
Annual number of publications on female CPP from 2005 to 2026. The dashed line indicates the linear regression trend fitted to complete calendar years 2005–2025 (*y* = 41.10*x* + 187.8, R^2^ = 0.9415). The year 2026 includes data through June 29 only and was excluded from the regression.

The most cited article was the ESHRE guideline on endometriosis management published in *Human Reproduction* in 2014 (total citations [TC] = 1582) [24]. The second most cited article was a clinical practice review on endometriosis published in the *New England Journal of Medicine* in 2010 (TC = 1545) [25]. The third most cited article addressed the origin and pathogenesis of epithelial ovarian cancer, published in the *American Journal of Surgical Pathology* in 2010 (TC = 1422) [26]. A complete list of the top 50 highly cited articles is provided in Supporting Table S1.

### 3.2. Analysis of Countries

As depicted in Figure 3A and summarized in Supporting Table S2, the USA emerged as the predominant contributor, with 2916 articles attributed to corresponding authors from the country (20.7% of total output), the highest number of publications with at least one author affiliated to the country (total publications, TP = 10,475), and the highest TC = 91,126, average citations = 31.3. China ranked second in corresponding‐author article count (*n* = 2,326, 16.5%) and in TP (7,212) but had a lower average citation rate (14.1). Italy ranked third in corresponding‐author article count (*n* = 1138) and achieved the second‐highest TC (36,087) with a notably high average citation rate (31.7). Among the top 20 countries, Belgium had the highest average citation rate (53.3) and the highest multiple country publication (MCP) ratio (48.7%), while Turkey had the lowest MCP ratio (2.3%), suggesting limited international collaboration. The United Kingdom demonstrated the second‐highest average citation rate (46.3) and a high MCP ratio (28.0%).

**FIGURE 3:**
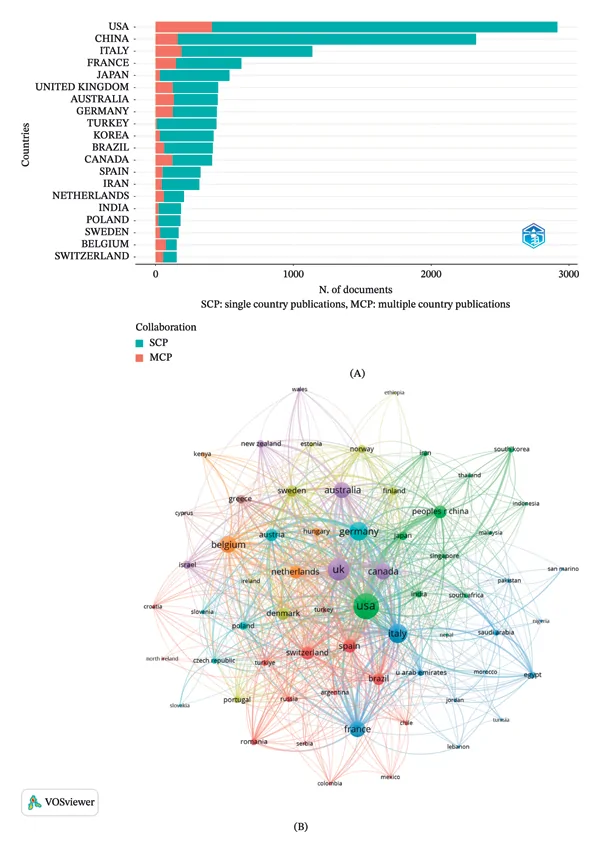
Country‐level analysis. (A) Distribution of corresponding authors’ publications by country. SCP: single country publications; MCP: multiple country publications. (B) Visualization map depicting international collaboration among countries.

The collaboration network map (Figure 3B) illustrated international cooperation patterns. Among the 64 countries with a minimum of 10 articles, the USA had the highest number of collaborations (1,507), followed by the UK (1,059) and Italy (794).

### 3.3. Analysis of Institutions

The top 10 institutions by article count are presented in Figure 4A. Harvard University led with 814 articles, followed by Assistance Publique–Hôpitaux de Paris (APHP, 659) and Université Paris Cité (629). Harvard University Medical Affiliates (550), the University of California System (543), Institut National de la Santé et de la Recherche Médicale (INSERM, 378), Mayo Clinic (303), Fudan University (297), the University of Genoa (273), and KU Leuven (264) completed the top ten.

**FIGURE 4:**
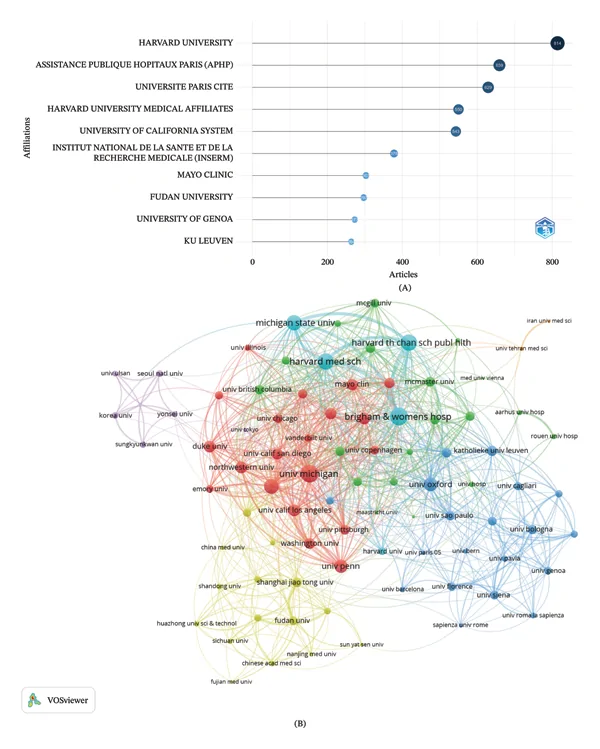
Institution‐level analysis. (A) Top 10 institutions by article count. Circle size represents article count; darker shades indicate higher ranks. (B) Visualization map depicting collaboration among institutions.

The institutional collaboration network (Figure 4B) revealed that, among 86 institutions with a minimum of 50 articles, Brigham and Women’s Hospital had the highest total link strength (276), followed by the University of Michigan (265) and Harvard Medical School (231). The institutional collaboration data are provided in Supporting Table S3.

### 3.4. Analysis of Journals

High‐impact journals ranked by h‐index are summarized in Supporting Table S4. *Fertility and Sterility* was the leading journal with the highest within‐dataset h‐index (94), the most publications (TP = 679, ranked 1st), and the highest TC (34,864), with an IF of 8.1 (Q1). *Human Reproduction* ranked second, with an h‐index of 91, 434 publications (ranked 3rd), and a TC of 22,492 (IF = 6.8, Q1). The *Journal of Minimally Invasive Gynecology* had the second highest publication count (TP = 506) but a lower h‐index of 47 (IF = 2.9, Q2). The *American Journal of Obstetrics and Gynecology* had the third highest TC (12,155) with an IF of 7.9 (Q1).

The journal co‐occurrence network comprised 48 journals with at least 50 occurrences (Figure 5A). *Fertility and Sterility* exhibited the highest total link strength (6,668), followed by *Human Reproduction* (6,020) and the *Journal of Minimally Invasive Gynecology* (2,777). The bibliographic coupling network (Figure 5B) similarly identified *Fertility and Sterility* as having the strongest coupling link strength (461,550), followed by *Human Reproduction* (358,651) and the *Journal of Minimally Invasive Gynecology* (216,218).

**FIGURE 5:**
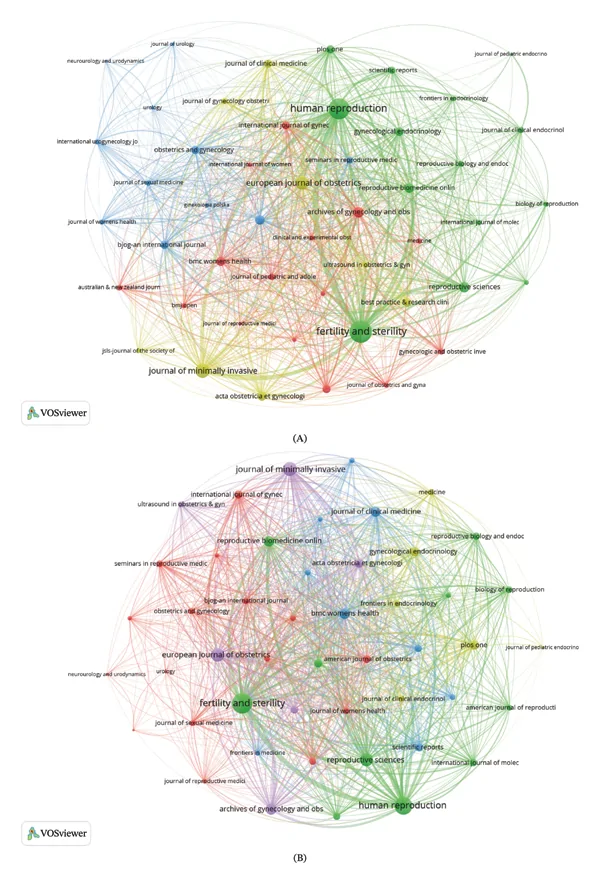
Journal‐level analysis. (A) Journal co‐occurrence network diagram. (B) Journal bibliographic coupling network diagram.

### 3.5. Analysis of Authors

Among the most influential authors ranked by within‐dataset h‐index, Chapron Charles led with the highest h‐index (35), a g‐index of 65, and 86 publications (TC = 4425). Vercellini Paolo ranked second (h‐index = 34, TP = 75, TC = 3793), and Taylor Hugh S. ranked third (h‐index = 33, TP = 71, TC = 4010). Giudice Linda C. achieved the highest TC (6,650) despite fewer publications (TP = 31), reflecting exceptionally high citation impact. Roman Horace had the highest publication count (TP = 92, ranked 1st) with an h‐index of 30. In the author collaboration network (Figure 6), among the 116 authors with at least 20 articles, Chapron Charles exhibited the strongest collaborative ties (total link strength = 244), followed by Seracchioli Renato (239) and Osuga Yutaka (235).

**FIGURE 6:**
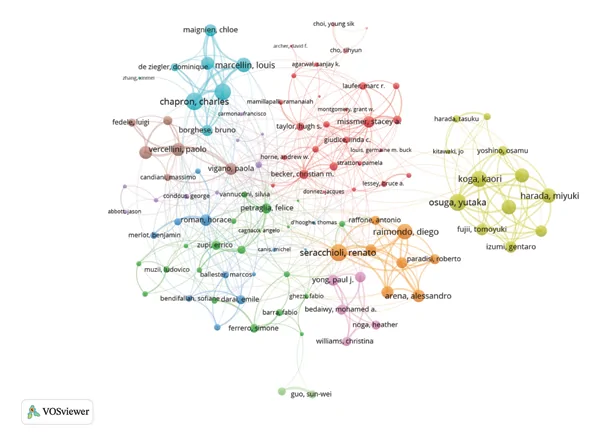
Visualization map depicting collaboration among authors.

### 3.6. Analysis of Keywords

Keyword co‐occurrence analysis identified 117 keywords with a minimum of 100 occurrences, organized into four clusters (Figure 7A). Cluster 1 (red, 40 items) encompassed clinical assessment and psychosocial dimensions, including terms such as “chronic pelvic pain,” “quality‐of‐life,” “prevalence,” “depression,” and “anxiety.” Cluster 2 (green, 36 items) centered on molecular pathogenesis and biological mechanisms, featuring keywords such as “expression,” “pathogenesis,” “inflammation,” “apoptosis,” and “peritoneal‐fluid.” Cluster 3 (blue, 32 items) focused on surgical management and disease classification, containing terms such as “surgery,” “diagnosis,” “management,” “laparoscopic excision,” and “deep endometriosis.” Cluster 4 (yellow, 9 items) addressed specific populations and conditions, including “adolescents,” “children,” “dysmenorrhea,” and “endometriosis.” The overlay visualization (Figure 7B) showed temporal trends in keyword emergence, with earlier keywords (2016–2017) centered on “diagnosis” and “surgery,” and more recent keywords (2018–2019) shifting toward “anxiety,” “depression,” and “health.”

**FIGURE 7:**
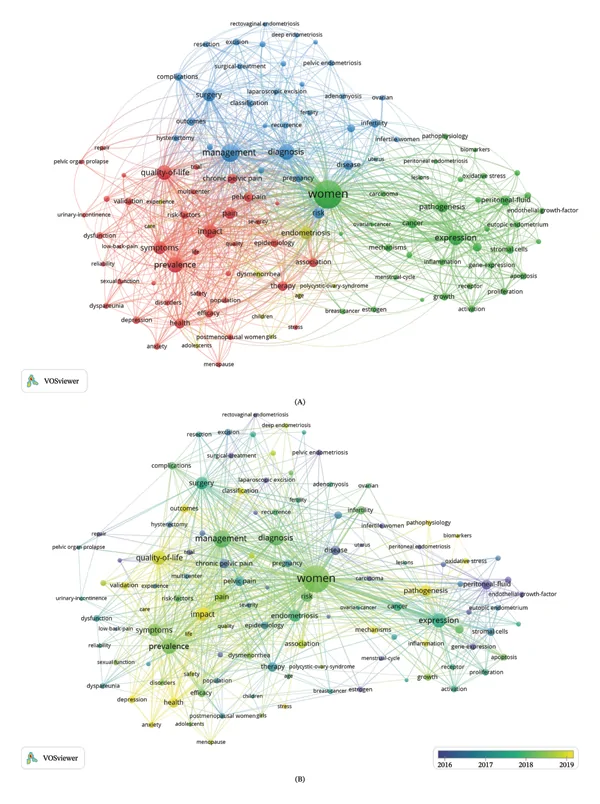
Keyword analysis. (A) Keyword co‐occurrence network with cluster visualization. (B) Overlay visualization of keyword co‐occurrence network showing temporal trends (color gradient indicates average publication year).

### 3.7. Analysis of Burst Keywords

Citation burst analysis identified the top 20 keywords with the strongest bursts over the study period (Figure 8). In the early period, “peritoneal fluid” exhibited the strongest burst (strength = 29.14, 2005–2013), followed by “pelvic endometriosis” (33.00, 2007–2017) and “trial” (28.26, 2010–2018). In the later period, “transvaginal ultrasound” (19.57, 2019–2024) and “anxiety” (22.19, 2020–2026) emerged as significant bursts. Most recently, “central precocious puberty” (18.95, 2023–2026), “case report” (17.43, 2023–2026), “life” (16.90, 2023–2026), and “Mendelian randomization” (17.20, 2024–2026) have been concentrated, indicating promising and rapidly evolving research directions.

**FIGURE 8:**
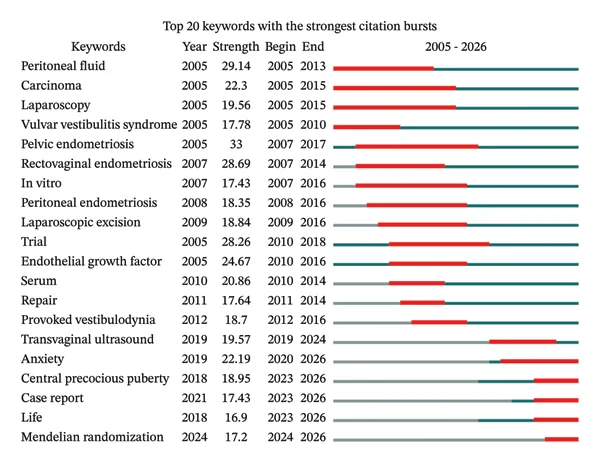
Top 20 keywords with the strongest citation bursts (CiteSpace).

## 4. Discussion

This bibliometric analysis characterized the global research landscape and evolving trends in the treatment of female CPP over the period from 2005 to 2026. A total of 14,110 articles from 1828 journals, involving 57,872 authors across 123 countries, were included. The annual publication output exhibited a consistent upward trajectory, rising from 242 articles in 2005 to 1223 in 2025, with an annual growth rate of 8.44%. Four distinct keyword clusters and multiple citation bursts were identified, collectively illustrating the thematic diversity and temporal evolution of this field. These findings provide a data‐driven overview of how research priorities in female CPP treatment have developed over the past 2 decades.

The USA was the leading contributor in terms of both publication volume and TCs, followed by China and Italy. While the USA and Italy exhibited comparable average citation rates, China had a substantially lower average citation count alongside a low international collaboration ratio. This pattern may reflect differences in collaborative engagement rather than research quality alone. Countries with higher MCP ratios, such as Belgium, the UK, Canada, and Australia, tended to achieve higher average citation rates, which is consistent with prior observations that international collaboration may facilitate broader dissemination and citation visibility [27]. Institutional analysis revealed that Harvard University, Assistance Publique Hopitaux de Paris, and Universite Paris Cite were the most productive institutions, while Brigham and Women’s Hospital and the University of Michigan demonstrated the strongest collaborative ties. The concentration of leading institutions in the USA and France aligns with the country‐level findings and may reflect sustained investment in reproductive health research infrastructure [28].

Among journals, *Fertility and Sterility* and *Human Reproduction* dominated both in publication volume and in co‐occurrence and bibliographic coupling network strength, suggesting that these two journals serve as central knowledge hubs for CPP‐related literature. Their high h‐indices and Q1 quartile rankings further underscore their role in shaping the intellectual foundation of this field [16]. The prominence of gynecology and obstetrics journals also indicates that CPP research remains primarily anchored within reproductive medicine, though the presence of journals such as *PLOS ONE* and the *Journal of Urology* suggests some degree of disciplinary cross‐pollination.

Author‐level analysis identified Chapron Charles, Vercellini Paolo, and Taylor Hugh S. as the most influential researchers based on h‐index. Notably, Giudice Linda C. achieved the highest TC count despite a relatively modest publication volume, indicating that certain contributions have had a disproportionate impact on the field. The most cited article in the dataset was the 2014 ESHRE guideline on the management of women with endometriosis, which has since been superseded by the 2022 update [9], which may reflect the centrality of standardized clinical guidance in shaping research directions. The high citation frequency of guideline documents and clinical practice reviews suggests that the field places considerable emphasis on translating evidence into practice frameworks.

### 4.1. Keyword Cluster Interpretation

Keyword co‐occurrence analysis identified four thematic clusters that together capture the major research dimensions in female CPP. Cluster 1 (clinical assessment and psychosocial dimensions) contained keywords such as “chronic pelvic pain,” “quality‐of‐life,” “depression,” “anxiety,” and “prevalence.” The prominence of psychological terms in this cluster is consistent with growing recognition that mental health comorbidities are prevalent among CPP patients. Recent meta‐analyses have estimated that approximately 40% of adults with chronic pain experience clinically significant depression and anxiety [29], and that women with CPP specifically exhibit elevated rates of anxiety disorders [30]. The co‐occurrence of “quality‐of‐life” with these psychological terms may suggest that research increasingly frames CPP not merely as a somatic condition but as a syndrome with substantial psychosocial dimensions, a perspective endorsed by current clinical guidelines recommending biopsychosocial approaches [8, 31].

Cluster 2 (molecular pathogenesis and biological mechanisms) featured keywords such as “expression,” “pathogenesis,” “peritoneal‐fluid,” “inflammation,” and “apoptosis.” This cluster indicates sustained attention to the molecular underpinnings of CPP‐related conditions, particularly endometriosis. The inclusion of terms such as “oxidative stress” and “stromal cells” suggests ongoing investigation into cellular and molecular pathways, which is consistent with the broader literature on endometriosis pathophysiology [32]. This mechanistic focus may complement clinical research by providing potential targets for therapeutic intervention.

Cluster 3 (surgical management and disease classification) included “surgery,” “diagnosis,” “management,” “laparoscopic excision,” and “deep endometriosis.” This cluster reflects the continued centrality of surgical approaches in CPP management. The co‐occurrence of “recurrence” with surgical keywords is notable, as postoperative recurrence remains a significant clinical challenge. Recent systematic reviews have reported recurrence as a persistent problem after conservative laparoscopic excision of ovarian endometrioma [33]. These reports are consistent with the keyword patterns observed here, which indicate that recurrence prevention remains a persistent research focus but provide no information on recurrence risk itself. The 2022 ESHRE guideline further recommends that laparoscopy is no longer the sole diagnostic gold standard, with transvaginal ultrasound (TVUS) recommended as a first‐line imaging modality [9]; the emergence of “transvaginal ultrasound” as a recent burst keyword reflects this growing research attention, whereas its diagnostic performance cannot be assessed by bibliometric methods.

Cluster 4 (specific populations and conditions) contained fewer items, including “adolescents,” “children,” “dysmenorrhea,” and “endometriosis.” The relatively small size of this cluster may suggest that age‐specific CPP research remains an emerging rather than established subfield, warranting further investigation into adolescent‐specific diagnostic and therapeutic strategies [34].

### 4.2. Burst Keyword Interpretation

Citation burst analysis provided temporal insight into evolving research emphases. Early bursts such as “peritoneal fluid” and “pelvic endometriosis” indicate that initial research momentum was oriented toward understanding the pathological environment associated with CPP. The subsequent emergence of “laparoscopic excision” and “trial” suggests a transition toward evaluating surgical interventions through controlled study designs. More recently, the burst of “anxiety” (2020–2026) and the overlay visualization showing temporal shifts toward psychological keywords are consistent with the growing body of evidence on psychosocial comorbidities in CPP [29, 30]. The appearance of “Mendelian randomization” as a burst keyword from 2024 onward may indicate increasing adoption of causal inference methodologies in CPP research, as several recent studies have applied this approach to explore genetic associations between endometriosis and various systemic outcomes [35, 36]. The burst of “central precocious puberty” from 2023 may reflect broadening research interest in hormonal and developmental factors relevant to pelvic pain conditions, though the precise connection warrants further exploration.

### 4.3. Clinical Implications From Bibliometric Trends

The temporal patterns observed in the keyword and burst analyses may carry implications for clinical practice and research prioritization. The co‐emergence of keywords related to psychological comorbidities, physical therapy, and central sensitization may suggest that CPP care is increasingly moving toward integrated, multidisciplinary models. This observation is consistent with recent systematic reviews reporting benefits of multimodal physical therapy for CPP [37] and with emerging evidence that interdisciplinary care programs may yield better outcomes than single‐discipline treatments [38]. Furthermore, the persistent appearance of surgical keywords alongside newer psychosocial terms suggests that research attention is increasingly distributed across gynecologic management, pelvic floor rehabilitation, and mental health‐informed care [39]. However, it should be emphasized that bibliometric data can identify research trends but cannot directly demonstrate the clinical effectiveness of any particular therapeutic approach.

### 4.4. Limitations

This study has several limitations that should be acknowledged. First, the reliance on the WoSCC as the sole data source may have led to the exclusion of relevant publications indexed in other databases such as Scopus, PubMed, or Embase. Second, the restriction to English‐language articles may have introduced language bias, potentially excluding valuable studies published in other languages. Third, although author, institution, and country names were standardized prior to analysis, residual name variations may persist and could affect ranking accuracy; variant entries such as those for Sapienza University of Rome were merged manually, but comparable variants in less frequently cited institutions cannot be excluded. Fourth, the expanded search strategy, while more comprehensive than a narrowly defined query, inevitably captures studies that span diagnostic and mechanistic domains beyond treatment per se, and the presence of diagnostic clusters in the results reflects this broader scope; the retrieved corpus should therefore be interpreted as the treatment‐related literature on female CPP broadly defined, rather than as a set of studies dealing exclusively with CPP. Fifth, the impact of database update timing on retrieved records cannot be entirely eliminated despite single‐day retrieval. Sixth, bibliometric analysis quantifies publication and citation patterns but does not assess clinical efficacy; high citation frequency does not necessarily equate to clinical importance or methodological quality. Seventh, citation bias, including self‐citation, language‐related citation preferences, and the inherent advantage of earlier publications in accumulating citations, may influence the observed rankings. Finally, the h‐index and related metrics may underrepresent recently emerging scholars due to citation lag.

## 5. Conclusion

This bibliometric analysis of 14,110 articles revealed sustained growth in female CPP treatment research from 2005 to 2026. Four thematic clusters and recent burst keywords suggest evolving emphasis toward psychosocial dimensions, integrated care approaches, and novel methodologies such as Mendelian randomization. These findings may inform future research prioritization and support the development of multidisciplinary CPP management strategies.

## Author Contributions

Conception and design: Zheng Hou and Haojie He. Administrative support: Haojie He and Ying Yao. Data analysis and interpretation: Zheng Hou, Sha Wang, and Yang Chen. Manuscript writing: All authors.

## Funding

This study was supported by a research grant (Grant No. 22‐486, awarded to Ying Yao) entitled “Effect of pelvic floor muscle training during pregnancy combined with postpartum pelvic floor rehabilitation on prevention of postpartum pelvic floor dysfunction disease.”

## Disclosure

All authors approved the final manuscript. The funders played no role in the design, conduct, or reporting of this study.

## Ethics Statement

The authors have nothing to report.

## Consent

The authors have nothing to report.

## Conflicts of Interest

The authors declare no conflicts of interest.

## Supporting Information

Additional supporting information can be found online in the Supporting Information section.

## Supporting information


**Supporting Information** Supporting Table S1. Top 50 Highly Cited Articles on Female CPP Treatment. Supporting Table S2. Publication and Citation Profiles of the Top 20 Contributing Countries. Supporting Table S3. Publication, Citation, and Collaboration Profiles of Institutions. Supporting Table S4. Bibliometric Indicators of the Top 20 High‐Impact Journals.

## Data Availability

All data generated or analyzed during this study are included in this article.
