# Supplementary material for: Global Research Landscape and Emerging Trends in the Treatment of Female Chronic Pelvic Pain: A Comprehensive Bibliometric Analysis (2005–2026)
Source: Pain Res Manag. 2026 Oct 18;2026:7732443. doi: 10.1155/prm/7732443 (PMC13678116; doi:10.1155/prm/7732443)
Supplement: Supplementary file 1 [file PRM-2026-7732443-s001.docx]

**Supplementary Table S1. Top 50 Highly Cited Articles on Female CPP Treatment**

| **Rank** | **Paper** | **DOI** | **Total Citations** | **TC per Year** | **Normalized TC** |
| --- | --- | --- | --- | --- | --- |
| 1 | Dunselman GAJ, 2014, Hum Reprod | 10.1093/humrep/det457 | 1582 | 121.69 | 46.04 |
| 2 | Giudice LC, 2010, New Engl J Med | 10.1056/NEJMcp1000274 | 1545 | 90.88 | 30.52 |
| 3 | Kurman RJ, 2010, Am J Surg Pathol | 10.1097/PAS.0b013e3181cf3d79 | 1422 | 83.65 | 28.09 |
| 4 | Kennedy S, 2005, Hum Reprod | 10.1093/humrep/dei135 | 1208 | 54.91 | 22.70 |
| 5 | Becker CM, 2022, Hum Reprod Open | 10.1093/hropen/hoac009 | 1204 | 240.80 | 80.61 |
| 6 | Burney RO, 2012, Fertil Steril | 10.1016/j.fertnstert.2012.06.029 | 1190 | 79.33 | 27.41 |
| 7 | Nnoaham KE, 2011, Fertil Steril | 10.1016/j.fertnstert.2011.05.090 | 1179 | 73.69 | 28.99 |
| 8 | Zondervan KT, 2018, Nat Rev Dis Primers | 10.1038/s41572-018-0008-5 | 1015 | 112.78 | 35.43 |
| 9 | Simoens S, 2012, Hum Reprod | 10.1093/humrep/des073 | 846 | 56.40 | 19.48 |
| 10 | Pearce CL, 2012, Lancet Oncol | 10.1016/S1470-2045(11)70404-1 | 668 | 44.53 | 15.38 |
| 11 | Burney RO, 2007, Endocrinology | 10.1210/en.2006-1692 | 600 | 30.00 | 13.36 |
| 12 | Agarwal SK, 2019, Am J Obstet Gynecol | 10.1016/j.ajog.2018.12.039 | 575 | 71.88 | 25.69 |
| 13 | Hanno PM, 2015, J Urology | 10.1016/j.juro.2015.01.086 | 573 | 47.75 | 17.06 |
| 14 | NA, 2012, Fertil Steril | 10.1016/j.fertnstert.2012.05.031 | 518 | 34.53 | 11.93 |
| 15 | Haggerty CL, 2010, J Infect Dis | 10.1086/652395 | 510 | 30.00 | 10.07 |
| 16 | Johnson NP, 2017, Hum Reprod | 10.1093/humrep/dew293 | 487 | 48.70 | 15.93 |
| 17 | Ait-Belgnaoui A, 2012, Psychoneuroendocrino | 10.1016/j.psyneuen.2012.03.024 | 485 | 32.33 | 11.17 |
| 18 | Ballard K, 2006, Fertil Steril | 10.1016/j.fertnstert.2006.04.054 | 479 | 22.81 | 9.03 |
| 19 | Hendren SK, 2005, Ann Surg | 10.1097/01.sla.0000171299.43954.ce | 468 | 21.27 | 8.80 |
| 20 | Meuleman C, 2009, Fertil Steril | 10.1016/j.fertnstert.2008.04.056 | 462 | 25.67 | 10.44 |
| 21 | NA, 2013, Menopause | 10.1097/gme.0b013e3182a122c2 | 450 | 32.14 | 12.39 |
| 22 | Johnson NP, 2013, Hum Reprod | 10.1093/humrep/det050 | 423 | 30.21 | 11.64 |
| 23 | Vercellini P, 2007, Hum Reprod | 10.1093/humrep/del339 | 422 | 21.10 | 9.39 |
| 24 | Taylor HS, 2017, New Engl J Med | 10.1056/NEJMoa1700089 | 406 | 40.60 | 13.28 |
| 25 | Latthe P, 2006, BMJ-Brit Med J | 10.1136/bmj.38748.697465.55 | 396 | 18.86 | 7.46 |
| 26 | Wentzensen N, 2016, J Clin Oncol | 10.1200/JCO.2016.66.8178 | 389 | 35.36 | 12.45 |
| 27 | Koninckx PR, 2012, Fertil Steril | 10.1016/j.fertnstert.2012.07.1061 | 386 | 25.73 | 8.89 |
| 28 | Gargett CE, 2009, Biol Reprod | 10.1095/biolreprod.108.075226 | 386 | 21.44 | 8.72 |
| 29 | De Graaff AA, 2013, Hum Reprod | 10.1093/humrep/det284 | 384 | 27.43 | 10.57 |
| 30 | Kingsberg SA, 2013, J Sex Med | 10.1111/jsm.12190 | 376 | 26.86 | 10.35 |
| 31 | Santoro N, 2015, Endocrin Metab Clin | 10.1016/j.ecl.2015.05.001 | 371 | 30.92 | 11.05 |
| 32 | Hudelist G, 2012, Hum Reprod | 10.1093/humrep/des316 | 368 | 24.53 | 8.48 |
| 33 | Falcone T, 2018, Obstet Gynecol | 10.1097/AOG.0000000000002469 | 367 | 40.78 | 12.81 |
| 34 | Anonymous, 2020, Menopause | 10.1097/GME.0000000000001609 | 363 | 51.86 | 16.77 |
| 35 | Facchin F, 2015, J Psychosom Obst Gyn | 10.3109/0167482X.2015.1074173 | 358 | 29.83 | 10.66 |
| 36 | Agarwal A, 2006, Curr Opin Obstet Gyn | 10.1097/01.gco.0000193003.58158.4e | 354 | 16.86 | 6.67 |
| 37 | Zimmermann A, 2012, BMC Womens Health | 10.1186/1472-6874-12-6 | 351 | 23.40 | 8.08 |
| 38 | Kvaskoff M, 2015, Hum Reprod Update | 10.1093/humupd/dmv013 | 345 | 28.75 | 10.27 |
| 39 | Sturdee DW, 2010, Climacteric | 10.3109/13697137.2010.522875 | 344 | 20.24 | 6.80 |
| 40 | Ballard KD, 2008, BJOG-Int J Obstet Gy | 10.1111/j.1471-0528.2008.01878.x | 338 | 17.79 | 8.61 |
| 41 | Lessey BA, 2019, Fertil Steril | 10.1016/j.fertnstert.2019.02.009 | 331 | 41.38 | 14.79 |
| 42 | Teague EMCO, 2009, Mol Endocrinol | 10.1210/me.2008-0387 | 324 | 18.00 | 7.32 |
| 43 | Bazot M, 2009, Fertil Steril | 10.1016/j.fertnstert.2008.09.005 | 321 | 17.83 | 7.25 |
| 44 | Nguyen JN, 2008, Obstet Gynecol | 10.1097/AOG.0b013e31816a2489 | 320 | 16.84 | 8.15 |
| 45 | Falconer H, 2015, JNCI-J Natl Cancer I | 10.1093/jnci/dju410 | 319 | 26.58 | 9.50 |
| 46 | Stratton P, 2011, Hum Reprod Update | 10.1093/humupd/dmq050 | 316 | 19.75 | 7.77 |
| 47 | Roseweir AK, 2009, J Neurosci | 10.1523/JNEUROSCI.5740-08.2009 | 296 | 16.44 | 6.69 |
| 48 | Merrill RM, 2008, Med Sci Monitor | — | 285 | 15.00 | 7.26 |
| 49 | Meuleman C, 2011, Hum Reprod Update | 10.1093/humupd/dmq057 | 276 | 17.25 | 6.79 |
| 50 | Naftalin J, 2012, Hum Reprod | 10.1093/humrep/des332 | 274 | 18.27 | 6.31 |

Note(s): TC per Year = total citations divided by the number of years since publication. Normalized TC = TC per Year normalized to the average citation rate of the corresponding publication year.

**Supplementary Table S2. Publication and Citation Profiles of the Top 20 Contributing Countries**

| **Country** | **Articles** | **Freq** | **SCP** | **MCP** | **MCP_Ratio** | **TP** | **TP_Rank** | **TC** | **TC_Rank** | **Average Citations** |
| --- | --- | --- | --- | --- | --- | --- | --- | --- | --- | --- |
| USA | 2916 | 0.207 | 2507 | 409 | 0.140 | 10475 | 1 | 91126 | 1 | 31.3 |
| China | 2326 | 0.165 | 2165 | 161 | 0.069 | 7212 | 2 | 32732 | 3 | 14.1 |
| Italy | 1138 | 0.081 | 950 | 188 | 0.165 | 4017 | 3 | 36087 | 2 | 31.7 |
| France | 623 | 0.044 | 473 | 150 | 0.241 | 2829 | 4 | 17895 | 5 | 28.7 |
| Japan | 537 | 0.038 | 504 | 33 | 0.061 | 1502 | 8 | 11765 | 8 | 21.9 |
| United Kingdom | 454 | 0.032 | 327 | 127 | 0.280 | 1764 | 5 | 21017 | 4 | 46.3 |
| Australia | 451 | 0.032 | 317 | 134 | 0.297 | 1749 | 7 | 12546 | 7 | 27.8 |
| Germany | 444 | 0.031 | 317 | 127 | 0.286 | 1756 | 6 | 8866 | 10 | 20.0 |
| Turkey | 442 | 0.031 | 432 | 10 | 0.023 | 1206 | 14 | 7972 | 13 | 18.0 |
| Korea | 421 | 0.030 | 388 | 33 | 0.078 | 1300 | 11 | 7518 | 14 | 17.9 |
| Brazil | 415 | 0.029 | 352 | 63 | 0.152 | 1234 | 13 | 9093 | 9 | 21.9 |
| Canada | 411 | 0.029 | 288 | 123 | 0.299 | 1499 | 9 | 13748 | 6 | 33.5 |
| Spain | 327 | 0.023 | 276 | 51 | 0.156 | 1343 | 10 | 6037 | 15 | 18.5 |
| Iran | 318 | 0.023 | 273 | 45 | 0.142 | 1260 | 12 | 5432 | 16 | 17.1 |
| Netherlands | 207 | 0.015 | 146 | 61 | 0.295 | 981 | 15 | 8334 | 11 | 40.3 |
| India | 185 | 0.013 | 162 | 23 | 0.124 | 536 | 20 | 2705 | 21 | 14.6 |
| Poland | 182 | 0.013 | 162 | 20 | 0.110 | 703 | 17 | 2321 | 24 | 12.8 |
| Sweden | 168 | 0.012 | 134 | 34 | 0.202 | 711 | 16 | 4884 | 17 | 29.1 |
| Belgium | 154 | 0.011 | 79 | 75 | 0.487 | 619 | 18 | 8201 | 12 | 53.3 |
| Switzerland | 154 | 0.011 | 99 | 55 | 0.357 | 552 | 19 | 3721 | 18 | 24.2 |

Note(s): Articles = number of articles by corresponding author's country; Freq = relative frequency of articles; SCP = single country publications; MCP = multiple country publications; MCP_Ratio = proportion of internationally co-authored publications; TP = total publications (including all co-authored contributions regardless of corresponding authorship); TP_Rank = rank by total publications; TC = total citations; TC_Rank = rank by total citations; Average Citations = TC divided by Articles.

**Supplementary Table S3. Publication, Citation, and Collaboration Profiles of Institutions**

| Rank | Institution | Documents | Citations | Total link strength |
| --- | --- | --- | --- | --- |
| 1 | Brigham & Womens Hosp | 115 | 6,811 | 276 |
| 2 | Univ Michigan | 141 | 4,800 | 265 |
| 3 | Harvard Med Sch | 124 | 3,377 | 231 |
| 4 | Harvard TH Chan Sch Publ Hlth | 66 | 3,993 | 219 |
| 5 | Michigan State Univ | 94 | 4,914 | 197 |
| 6 | Univ Washington | 104 | 3,898 | 186 |
| 7 | Univ Oxford | 114 | 11,675 | 157 |
| 8 | Univ Penn | 72 | 3,325 | 147 |
| 9 | Univ Calif Los Angeles | 65 | 2,515 | 119 |
| 10 | Northwestern Univ | 82 | 3,403 | 115 |
| 11 | Univ Calif San Francisco | 116 | 9,548 | 114 |
| 12 | Duke Univ | 59 | 1,869 | 114 |
| 13 | Stanford Univ | 94 | 4,124 | 111 |
| 14 | Mayo Clin | 129 | 3,473 | 107 |
| 15 | Univ Calif San Diego | 91 | 2,665 | 105 |
| 16 | Washington Univ | 57 | 1,809 | 98 |
| 17 | Cleveland Clin | 83 | 4,019 | 86 |
| 18 | Katholieke Univ Leuven | 70 | 6,144 | 86 |
| 19 | Shanghai Jiao Tong Univ | 94 | 1,953 | 80 |
| 20 | McMaster Univ | 59 | 2,237 | 80 |
| 21 | Univ Pittsburgh | 76 | 2,674 | 79 |
| 22 | Univ Toronto | 71 | 2,220 | 79 |
| 23 | Univ Sao Paulo | 156 | 5,227 | 77 |
| 24 | Univ British Columbia | 95 | 3,127 | 77 |
| 25 | Univ Queensland | 76 | 2,572 | 76 |
| 26 | Univ Edinburgh | 59 | 6,017 | 74 |
| 27 | Univ Bologna | 112 | 3,091 | 73 |
| 28 | Univ Sydney | 103 | 2,512 | 73 |
| 29 | Univ Utah | 56 | 3,092 | 71 |
| 30 | Univ Adelaide | 82 | 3,024 | 70 |
| 31 | Fudan Univ | 171 | 4,395 | 69 |
| 32 | McGill Univ | 90 | 2,944 | 69 |
| 33 | Monash Univ | 87 | 4,442 | 69 |
| 34 | Univ Milan | 153 | 7,823 | 67 |
| 35 | Univ Siena | 82 | 3,623 | 63 |
| 36 | Univ Copenhagen | 53 | 2,866 | 63 |
| 37 | Univ Chicago | 50 | 983 | 62 |
| 38 | Univ N Carolina | 66 | 3,892 | 60 |
| 39 | Univ Cagliari | 59 | 1,976 | 60 |
| 40 | Yale Univ | 87 | 4,680 | 59 |
| 41 | Queens Univ | 80 | 3,486 | 58 |
| 42 | Univ Florence | 68 | 1,697 | 56 |
| 43 | Harvard Univ | 65 | 3,587 | 53 |
| 44 | Yale Sch Med | 62 | 2,885 | 53 |
| 45 | Emory Univ | 50 | 1,436 | 53 |
| 46 | Vanderbilt Univ | 55 | 1,163 | 51 |
| 47 | Univ Melbourne | 84 | 2,343 | 50 |
| 48 | Univ Genoa | 105 | 4,148 | 49 |
| 49 | Capital Med Univ | 95 | 1,382 | 48 |
| 50 | Sapienza Univ Rome (merged) | 104 | 3,193 | 47 |
| 51 | Univ Cattolica Sacro Cuore | 74 | 3,413 | 47 |
| 52 | Univ Naples Federico II | 70 | 1,530 | 45 |
| 53 | Korea Univ | 52 | 955 | 41 |
| 54 | Peking Univ | 64 | 1,234 | 40 |
| 55 | Univ Montreal | 57 | 2,174 | 39 |
| 56 | Zhejiang Univ | 147 | 2,606 | 38 |
| 57 | Seoul Natl Univ | 62 | 1,846 | 37 |
| 58 | Karolinska Inst | 120 | 5,323 | 36 |
| 59 | Univ Illinois | 62 | 2,474 | 36 |
| 60 | Univ Pavia | 51 | 3,057 | 35 |
| 61 | Yonsei Univ | 60 | 1,366 | 34 |
| 62 | Sungkyunkwan Univ | 54 | 1,306 | 32 |
| 63 | Chinese Acad Med Sci | 54 | 1,180 | 28 |
| 64 | Shandong Univ | 74 | 1,581 | 27 |
| 65 | Univ Ulsan | 53 | 1,128 | 27 |
| 66 | Univ Barcelona | 63 | 1,259 | 26 |
| 67 | Sichuan Univ | 71 | 1,011 | 25 |
| 68 | Huazhong Univ Sci & Technol | 71 | 1,233 | 25 |
| 69 | Univ Hosp | 53 | 1,177 | 25 |
| 70 | Aarhus Univ Hosp | 75 | 1,959 | 24 |
| 71 | Rouen Univ Hosp | 79 | 2,309 | 23 |
| 72 | Baylor Coll Med | 67 | 1,471 | 23 |
| 73 | Univ Paris 05 | 88 | 5,054 | 20 |
| 74 | Tel Aviv Univ | 82 | 1,701 | 20 |
| 75 | Univ Bern | 62 | 2,882 | 20 |
| 76 | China Med Univ | 84 | 989 | 17 |
| 77 | Nanjing Med Univ | 67 | 660 | 17 |
| 78 | Univ Tokyo | 94 | 3,972 | 15 |
| 79 | Univ Tehran Med Sci | 84 | 1,194 | 15 |
| 80 | Sun Yat Sen Univ | 75 | 888 | 13 |
| 81 | Maastricht Univ | 50 | 2,870 | 13 |
| 82 | Iran Univ Med Sci | 51 | 895 | 9 |
| 83 | Fujian Med Univ | 51 | 400 | 9 |
| 84 | Univ Oslo | 50 | 1,955 | 8 |
| 85 | Med Univ Vienna | 52 | 1,227 | 6 |

Note(s): Documents = total number of articles published by the institution; Citations = total citations received; Total Link Strength = strength of collaborative ties with other institutions in the co-authorship network (VOSviewer). Institutions are ranked by Total Link Strength in descending order.

**Supplementary Table S4. Bibliometric Indicators of the Top 20 High-Impact Journals**

| **Rank** | **Journal** | **H_index** | **IF** | **JCR** | **g-index** | **m-index** | **TP** | **TP_Rank** | **TC** | **TC_Rank** | **PY_start** |
| --- | --- | --- | --- | --- | --- | --- | --- | --- | --- | --- | --- |
| 1 | Fertility and Sterility | 94 | 8.1 | Q1 | 143 | 4.273 | 679 | 1 | 34864 | 1 | 2005 |
| 2 | Human Reproduction | 91 | 6.8 | Q1 | 144 | 4.136 | 434 | 3 | 22492 | 2 | 2005 |
| 3 | Journal of Minimally Invasive Gynecology | 47 | 2.9 | Q2 | 60 | 2.136 | 506 | 2 | 5770 | 8 | 2005 |
| 4 | American Journal of Obstetrics and Gynecology | 46 | 7.9 | Q1 | 74 | 2.091 | 139 | 17 | 12155 | 3 | 2005 |
| 5 | Journal of Clinical Endocrinology & Metabolism | 46 | 5.4 | Q1 | 65 | 2.091 | 85 | 31 | 6027 | 7 | 2005 |
| 6 | Journal of Sexual Medicine | 45 | 3.6 | Q1 | 68 | 2.045 | 182 | 11 | 4042 | 10 | 2005 |
| 7 | Obstetrics and Gynecology | 43 | 5.1 | Q1 | 73 | 1.955 | 129 | 19 | 11224 | 4 | 2005 |
| 8 | European Journal of Obstetrics & Gynecology and Reproductive Biology | 42 | 2.3 | Q2 | 62 | 1.909 | 358 | 4 | 6062 | 6 | 2005 |
| 9 | Reproductive Sciences | 36 | 2.8 | Q2 | 54 | 1.800 | 217 | 7 | 3419 | 19 | 2007 |
| 10 | BJOG-An International Journal of Obstetrics and Gynaecology | 35 | 4.8 | Q1 | 66 | 1.591 | 84 | 32 | 3489 | 17 | 2005 |
| 11 | Journal of Urology | 35 | 9.7 | Q1 | 53 | 1.591 | 53 | 45 | 3572 | 16 | 2005 |
| 12 | Archives of Gynecology and Obstetrics | 34 | 2.6 | Q2 | 47 | 1.789 | 247 | 5 | 3438 | 18 | 2008 |
| 13 | International Urogynecology Journal | 34 | 2.0 | Q3 | 51 | 1.545 | 184 | 10 | 3396 | 20 | 2005 |
| 14 | PLOS ONE | 34 | 2.8 | Q2 | 47 | 1.889 | 170 | 14 | 3668 | 14 | 2009 |
| 15 | Acta Obstetricia et Gynecologica Scandinavica | 33 | 3.5 | Q1 | 48 | 1.500 | 149 | 16 | 3926 | 11 | 2005 |
| 16 | Gynecological Endocrinology | 32 | 3.0 | Q2 | 45 | 1.455 | 189 | 9 | 2376 | 29 | 2005 |
| 17 | Journal of Pediatric and Adolescent Gynecology | 32 | 2.0 | Q2 | 50 | 1.600 | 127 | 21 | 1417 | 49 | 2007 |
| 18 | Reproductive BioMedicine Online | 31 | 3.9 | Q1 | 50 | 1.409 | 137 | 18 | 2808 | 22 | 2005 |
| 19 | International Journal of Gynecology & Obstetrics | 30 | 2.7 | Q2 | 47 | 1.364 | 180 | 13 | 2685 | 23 | 2005 |
| 20 | Journal of Obstetrics and Gynaecology Research | 29 | 1.7 | Q3 | 43 | 1.318 | 201 | 8 | 1664 | 43 | 2005 |

Note(s): H_index = h-index of the journal, measuring both productivity and citation impact of publications; IF = Journal Impact Factor (2025), indicating the average number of citations to recent articles; JCR = Journal Citation Reports quartile ranking (Q1 = top 25%, Q2 = 25%–50%, Q3 = 50%–75%, Q4 = bottom 25%); g-index = gives greater weight to highly cited articles; m-index = h-index divided by the number of years since the first published paper in the dataset, indicating sustained scholarly consistency; TP = total publications; TP_Rank = rank by total publications; TC = total citations; TC_Rank = rank by total citations; PY_start = first publication year in the dataset.
